# Association between Footwear Use and Neglected Tropical Diseases: A Systematic Review and Meta-Analysis

**DOI:** 10.1371/journal.pntd.0003285

**Published:** 2014-11-13

**Authors:** Sara Tomczyk, Kebede Deribe, Simon J. Brooker, Hannah Clark, Khizar Rafique, Stefanie Knopp, Jürg Utzinger, Gail Davey

**Affiliations:** 1 Institute of Tropical Medicine, Antwerp, Belgium; 2 Brighton & Sussex Medical School, Brighton, United Kingdom; 3 School of Public Health, Addis Ababa University, Addis Ababa, Ethiopia; 4 London School of Hygiene and Tropical Medicine, London, United Kingdom; 5 Wolfson Wellcome Biomedical Laboratories, Department of Life Sciences, Natural History Museum, London, United Kingdom; 6 Department of Epidemiology and Public Health, Swiss Tropical and Public Health Institute, Basel, Switzerland; 7 University of Basel, Basel, Switzerland; George Washington University, United States of America

## Abstract

**Background:**

The control of neglected tropical diseases (NTDs) has primarily focused on preventive chemotherapy and case management. Less attention has been placed on the role of ensuring access to adequate water, sanitation, and hygiene and personal preventive measures in reducing exposure to infection. Our aim was to assess whether footwear use was associated with a lower risk of selected NTDs.

**Methodology:**

We conducted a systematic review and meta-analysis to assess the association between footwear use and infection or disease for those NTDs for which the route of transmission or occurrence may be through the feet. We included Buruli ulcer, cutaneous larva migrans (CLM), leptospirosis, mycetoma, myiasis, podoconiosis, snakebite, tungiasis, and soil-transmitted helminth (STH) infections, particularly hookworm infection and strongyloidiasis. We searched Medline, Embase, Cochrane, Web of Science, CINAHL Plus, and Popline databases, contacted experts, and hand-searched reference lists for eligible studies. The search was conducted in English without language, publication status, or date restrictions up to January 2014. Studies were eligible for inclusion if they reported a measure of the association between footwear use and the risk of each NTD. Publication bias was assessed using funnel plots. Descriptive study characteristics and methodological quality of the included studies were summarized. For each study outcome, both outcome and exposure data were abstracted and crude and adjusted effect estimates presented. Individual and summary odds ratio (OR) estimates and corresponding 95% confidence intervals (CIs) were calculated as a measure of intervention effect, using random effects meta-analyses.

**Principal Findings:**

Among the 427 studies screened, 53 met our inclusion criteria. Footwear use was significantly associated with a lower odds of infection of Buruli ulcer (OR = 0.15; 95% CI: 0.08–0.29), CLM (OR = 0.24; 95% CI: 0.06–0.96), tungiasis (OR = 0.42; 95% CI: 0.26–0.70), hookworm infection (OR = 0.48; 95% CI: 0.37–0.61), any STH infection (OR = 0.57; 95% CI: 0.39–0.84), strongyloidiasis (OR = 0.56; 95% CI: 0.38–0.83), and leptospirosis (OR = 0.59; 95% CI: 0.37–0.94). No significant association between footwear use and podoconiosis (OR = 0.63; 95% CI: 0.38–1.05) was found and no data were available for mycetoma, myiasis, and snakebite. The main limitations were evidence of heterogeneity and poor study quality inherent to the observational studies included.

**Conclusions/Significance:**

Our results show that footwear use was associated with a lower odds of several different NTDs. Access to footwear should be prioritized alongside existing NTD interventions to ensure a lasting reduction of multiple NTDs and to accelerate their control and elimination.

**Protocol Registration:**

PROSPERO International prospective register of systematic reviews CRD42012003338

## Author Summary

Consistent use of footwear may help in preventing or slowing down the progression of many neglected tropical diseases (NTDs). We conducted a systematic review and meta-analysis to assess the association between footwear use and infection or disease for those NTDs for which the route of transmission or occurrence may be through the feet. We found that footwear use reduces the risk of Buruli ulcer, tungiasis, hookworm, any STH infection, strongyloidiasis, and leptospirosis. No significant association between footwear use and podoconiosis was found and no data were available for mycetoma, myiasis or snakebite. We recommend that access to footwear should be prioritized alongside existing NTD interventions to ensure a lasting reduction of multiple NTDs and to accelerate their control and elimination.

## Introduction

Neglected tropical diseases (NTDs) are caused by a variety of pathogens, such as parasites (e.g., ectoparasites, helminths, and protozoa), fungi, bacteria, and viruses, primarily found in the tropical and subtropical regions of the world [1]. NTDs mainly occur in rural and deprived urban areas of low- and middle-income countries, where they may exacerbate poverty by contributing to significant morbidity and mortality, impairing development, and limiting productivity [1], [2]. They have multiple routes of transmission and a single intervention alone is unlikely to have major sustained impact. Population-based chemotherapy is currently the mainstay of the control of various NTDs caused by helminths (e.g., lymphatic filariasis, schistosomiasis, and soil-transmitted helminth (STH) infections) and some bacterial infections (e.g., trachoma) [3], [4]. More recently, attention has been given to water, sanitation, and hygiene (WASH) as an effective and sustainable measure for NTD control [5]–[7]. WASH interventions such as face washing to prevent trachoma, or hand washing to prevent diarrheal diseases and STH infection have been well-studied [8]–[10]. However, less attention has focused on other personal preventive measures to reduce exposure to infection, such as the use of footwear. Some NTDs may be transmitted or occur through the feet, and hence, footwear could prevent this exposure. To our knowledge, there has not yet been a systematic review of the evidence to assess the role of footwear use among these NTDs [8]–[12].

There is continued debate over the role of footwear use as an additional measure of NTD control [13]–[15]. Some studies have highlighted that footwear use could reduce infection with hookworm caused by *Necatoramericanus* and/or *Ancylostoma duodenale* (which is also orally infective), but such studies are often cross-sectional and should be interpreted with caution [15]–[17]. Other experts argue that decreases in the burden of hookworm disease are based on large-scale administration of deworming drugs (a strategy termed preventive chemotherapy), socioeconomic development, and improved access to WASH rather than widespread footwear use, while newer evidence indicates that the burden from hookworm disease has not changed significantly over the past 20 years [13], [18]. Furthermore, the lack of adequate change in hookworm disease burden might be due to the overwhelming focus on preventive chemotherapy over the last few decades and less emphasis on other interventions [5]. In the case of podoconiosis (non-filarial elephantiasis), footwear use is currently promoted as a prevention tool, since current evidence suggests that it is caused by barefoot exposure to red clay soil from volcanic rocks [19]. Other studies and anecdotal evidence have additionally suggested that footwear use may prevent Buruli ulcer, cutaneous larva migrans (CLM), leptospirosis, mycetoma (fungal eumycetoma and bacterial actinomycetoma), myiasis, snakebite, strongyloidiasis, and tungiasis [20], [21].

Here, we first identified those NTDs for which the use of footwear might have a potential impact on the risk of infection and disease, based on an understanding of disease etiology and transmission. We next conducted a systematic review and series of meta-analyses of the association between footwear use and the risk of a range of NTDs.

## Methods

NTDs were selected to be included in the study based on disease etiology and potential for infection through the feet and thus prevention using footwear (Table 1). A systematic literature review protocol strategy was developed based on the ‘Preferred Reporting Items for Systematic reviews and Meta-Analyses' (PRISMA) checklist (e.g., protocol and registration, eligibility criteria, information sources, searching, study selection, data collection process, data items, risk of bias in individual studies, summary measures, synthesis of results, risk of bias across studies, and additional analyses (see: Checklist S1). This protocol is available at the National Institute for Health Research PROSPERO International prospective register of systematic reviews (identifier: CRD42012003338) (see Protocol S1).

**Table 1:** Overview of included neglected tropical diseases (NTDs) in the current systematic review and meta-analysis.

#	Disease	Aetiology	Search Terms+
1	Buruli ulcer	*Mycobacterium ulcerans*: precise transmission unknown but may be associated with insect bites to exposed skin such as feet	exp Buruli Ulcer OR exp, *Mycobacterium ulcerans* OR exp, mycobacterium infections, nontuberculous OR buruli ulcer* OR mycobacterium ulceran* OR Bairnsdale ulcer OR Daintree ulcer
2	Podoconiosis	Geochemical non-filarial elephantiasis, Transmission associated with long term barefoot exposure to red clay soil	Podoconiosis OR non-filarial elephantiasis OR mossy foot
3	Any soil-transmitted helminth (STH) infection, including hookworm	*Ascaris lumbricoides: Trichuris trichiura* and hookworm, intestinal parasites which produce eggs passed in feces, transmission by ingestion from contaminated hands or utensils or penetration of skin by larvae (i.e., if feet are exposed to contaminated soil)	Soil-transmitted helminth* OR soil transmitted helminth* OR intestinal worm* OR exphelminth OR expHelminthiasis
4	Hookworm infection	*Necatoramericanus* and *Ancylostoma duodenale*, transmission by penetration of skin by larvae (i.e., if feet are exposed to contaminated soil)	Exp hookworm infections OR expancylostomatoidea OR expancylostoma OR necator
5	Strongyloidiasis	*Strongyloides stercoralis*: type of STH, which produces eggs that hatch into larvae passed in feces and transmission by penetration of skin by larvae(i.e., if feet are exposed to contaminated soil)	Strongyloid* OR exp strongyloides stercoralis OR exp Stronyloidiasis OR exp strongyloides OR round?worm
6	Cutaneous larva migrans	*Ancylostoma braziliense, A. ceylanicum* and other zoonotic hookworms: zoonotic intestinal parasite living in cats and dogs, which produce eggs passed in their feces, transmission by penetration of skin by larvae (i.e., if feet are exposed to contaminated soil)	Exp larva migrans OR cutaneous larva migran* OR creeping eruption OR ground itch OR sandworm* OR plumber's itch OR zoonotic hookworm OR ancylostoma braziliense OR uncinaria stenocephala OR ancylostoma caninum OR exp ancylostoma
7	Leptospirosis	*Leptospira interrogans: b*acteria passed in urine, *t*ransmission by direct contact through the mucous membranes of the mouth, nose, and eyes, or through cuts and abrasions on the skin (i.e., if feet are exposed to contaminated soil)	Exp leptospirosis OR weil's syndrome OR weil disease OR canicola fever OR canefield fever OR nanukayami fever OR 7-day fever OR Rat Catcher's Yellows OR Fort Bragg Fever OR black jaundice OR Pretibial fever OR Leptospira OR Icterohemorrhagic fever OR Swineherd's disease OR Rice-field fever OR Cane-cutter fever OR Swamp fever OR Mud fever OR Hemorrhagic jaundice OR Stuttgart disease
8	Tungiasis	*Tunga penetrans: e*ctoparasite on the sand flea, transmission by penetration of skin by sand fleas (i.e., if feet are exposed to contaminated sand)	Exptunga OR Tungapenetrans OR jigger* OR sandflea OR expTungiasis OR Pico OR chigoe flea OR suthi
9	Myiasis	*Dermatobiahominis, Cordylobiaanthropophaga* and others: parasite transmitted on a fly larva (and potentially through blood-sucking vectors such as mosquitos), transmission by penetration of skin by larvae(i.e., if feet are exposed to contaminated soil)	Exp myiasis OR dermatobiahominis OR chrysomabezziana OR cordylobiaanthropophaga OR flystrike OR blowfly strike OR fly-blown
10	Snakebite	Venomous snakes: envenoming, transmission associated with snake bites to exposed skin (i.e., on feet)	Exp snake bite OR exp antivenins OR snakebite* OR exp venoms OR envenoming OR snake poison
11	Mycetoma	Eumycetoma: *Madurellamycetomatis, Pseudallescheriaboydii* (and other fungi), Actinomycetoma: *Nocardia* spp., *Streptomyces* spp., *Actinomadura* spp. (and other aerobic actinomycetes), certain fungi or bacteria, transmission probably by entering the body into the subcutaneous tissue through minor trauma, often through the foot	Madura Foot OR expmycetoma OR eumycetoma* OR mycetomapedis OR actinomycetoma*

+Additional search terms for intervention (exp shoes OR shoe* OR footwear* OR boots OR sandals OR footgear OR exp primary prevention) and all NTDs (exp neglected diseases OR neglected tropical disease* OR NTD* OR exp tropical disease).

A total of 92 known medical and colloquial disease names (see Table 1) were included in a comprehensive list of key search terms. Six terms related to footwear were also included: shoe, footwear, boot, sandal, footgear, or primary prevention. Relevant databases were searched from using these terms, including Medline (coverage from 1950), Embase (coverage from 1947), Cochrane (coverage from 2003), Web of Science (coverage from 1900), CINAHL Plus (coverage from 1937), Popline (coverage from 1970), British Library for Development Studies (coverage from 1987), ELDIS (coverage date unavailable), EPPI-Centre (coverage from 2004), WHO Library (coverage from 1948), and PAHO Library Catalogue (coverage from 1902). The search was conducted from January 1, 2013 to December 31, 2014. Experts in selected NTD areas were contacted for further citation recommendations relevant to the research question. The Brighton and Sussex Medical School (BSMS) Library was consulted for assistance with article retrieval through online databases or manual journal searching. The reference lists of all identified manuscripts were also reviewed for additional citations. Manuscripts in foreign languages (namely, French, Spanish, and Russian) were translated by investigators. No other foreign language articles were identified through this search. When potentially eligible studies did not provide sufficient data in the manuscript, authors were contacted and asked if they would be willing to provide additional data. To this end, additional data were received from authors of five studies [22]–[26].

Pre-defined eligibility criteria included: (i) all intervention and observational study designs; (ii) all study settings; (iii) all ages; (iv) all types of footwear exposures; (v) prevalence or incidence estimates of infection and/or disease outcomes; (vi) all published manuscripts and grey literature; (vii) all publication dates; and (viii) all languages. Observational studies were included because it was hypothesized that few randomized controlled trials (RCTs) had been conducted to answer the research question. Abstracts of identified studies were reviewed before appraisal of full manuscripts when possible. If a study did not explicitly investigate the association between footwear use and any of the target NTDs or did not meet the eligibility criteria, it was excluded. Decisions on inclusion were reached by the consensus of independent screenings conducted by two investigators (ST and KD).

A standardized Excel data extraction form was developed based on the PRISMA statement [27] and used to record the following information: study ID, author, title, journal, publication year, type of literature, research question, study design, study setting, outcome, follow-up, sample size, number of cases, descriptive case data (e.g., age, sex, and proportion wearing footwear), descriptive control data (e.g., age, sex, and proportion wearing footwear), crude and adjusted effect estimates of footwear use on disease, including 95% confidence intervals (CIs) and p-values, and study quality ratings. The methodological quality of studies was assessed by the same investigators. According to a pre-defined scale, the following question was assessed, stating “Were the following items reported?”: (i) study population (e.g., social-ecological characteristics); (ii) selection of participants (i.e., random or convenience); (iii) sample size calculation; (iv) method of measuring footwear use and presence of NTDs; and (v) estimates adjusted for confounding. These questions were scored on a yes/no basis and proportions answering yes to each question were described to assess study quality and risk of bias in and across individual studies. None of the investigators on this review assessed the study quality of their own primary studies.

All primary data and quality ratings were extracted from identified manuscripts. STATA version 12.0 (College Station, TX, United States of America) was used to summarize the data descriptively. RevMan version 5.2 and its generic variance format was used to generate individual forest plots according to primary NTD outcomes [28].We entered odds ratio (OR) estimates of footwear use on a logarithmic scale and standard errors (calculated from 95% CIs). An adjusted OR was used if provided in the manuscript. A few studies only provided raw outcome and exposure data, so we calculated a crude OR in these cases. All calculations and data used are detailed in the footnotes of each figure. A random-effects model in RevMan was then utilized to produce individual study ORs and 95% CIs and to consider a pooled summary effect estimate (using random effects to address potential heterogeneity). Heterogeneity was assessed by the *I* ^2^ test with values greater than 50% representing moderate-to-severe heterogeneity.

## Results

**Figure 1:**
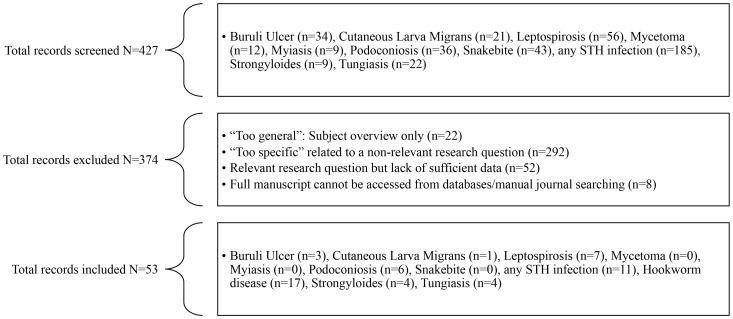
Flow diagram of eligibility and inclusion/exclusion procedures.

The electronic searches generated 427 citations and abstracts. These were screened and 374 were excluded for a range of reasons (Figure 1). We included 53 sources: Buruli ulcer (n = 3), CLM (n = 1), leptospirosis (n = 7), podoconiosis (n = 6), any STH infections (n = 11), hookworm infection (n = 17), strongyloidiasis (n = 4), and tungiasis (n = 4). No data were found to quantify the association between footwear use and mycetoma, myiasis, and snakebite. Type of source included 50 journal manuscripts (94.3%), two unpublished pieces of work (3.8%), and one book excerpt (1.9%). Information describing the studies included are summarized in Table 2, including study design, publication year, country and outcome. We identified a total of 40 cross-sectional studies (75.4%), eight case-control studies (15.1%), three cohort studies (5.7%), and two RCTs (3.8%). The median publication year was 2003 (range: 1950–2014). Geographically, 29 studies were conducted in Africa (54.7%), 12 in Asia (22.6%), 11 in the Americas (20.8%), and one in Europe (1.9%). The median sample size was 366 individuals (range: 59–129,959). Among the 11 studies with known follow-up periods, the median follow-up time was 12 months (range: 2.5 months to 7 years). Descriptive results by individual studies including sample size, median age, and proportion of females are shown in Table 2. A summary of descriptive results by outcome, including study quality results, are provided in Table 3.

**Table 2:** Included studies: Time, place, design of study, and descriptive results in the current systematic review and meta-analysis.

						With disease			Without disease		
ID	Author	Pub. Year	Study design	Country	Outcome	N	Age median	Female n (%)	N	Age median	Female n (%)
1	Landier et al. [44]	2011	Case-control	Cameroon	Buruli Ulcer	77	14.0	37 (48)	153	13.0	74 (48)
2	Marston et al. [84]	1995	Case-control	Côte d'Ivoire	Buruli Ulcer	46	16.0*	26 (57)	90	21.4*	45 (50)
3	Raghunathan et al. [45]	2005	Case-control	Ghana	Buruli Ulcer	116	12.0	62 (53)	116	11.5	57 (49)
4	Trembley et al. [20]	2000	Cross-sec.	Barbados	CLM	32	36.9*	+	94	41.2*	+
5	Bovet et al. [51]	1999	Case-control	Seychelles	Leptospirosis	125	39.0*	20 (16)	125	39.5*	20 (16)
6	Douglin et al. [85]	1997	Case-control	Barbados	Leptospirosis	22	30.8*	8 (36)	38	31.3*	23 (61)
7	Johnson et al. [46]	2004	Cross-sec.	Peru	Leptospirosis	182	29.0	105 (58)	466	29.0	251 (54)
8	Lacerda et al. [52]	2008	Cross-sec.	Brazil	Leptospirosis	44	26.0*	19 (43)	246	28.7*	120 (49)
9	Leal-Castellanos et al. [47]	2003	Cross-sec.	Mexico	Leptospirosis	441	40.8*	341 (77)	728	40.2*	605 (83)
10	Phraisuwan et al. [48]	2002	Case-control	Thailand	Leptospirosis	43	35.0	16 (37)	61	40.0	33 (54)
11	Sulong et al. [49]	2011	Cross-sec.	Malaysia	Leptospirosis	73	41.7*	0 (0)	223	42.2*	0 (0)
12	Sanchez et al. [55]	2001	Cohort	Spain	Strongyloidiasis	20	68.8*	1 (5)	132	66.0*	31 (23)
13	Steinmann et al. [54]	2007	Cross-sec.	China	Strongyloidiasis	21	29.0*	6 (29)	159	25.5*	92 (58)
14	Knopp et al. [22]	2010	Cross-sec.	Tanzania	Strongyloidiasis	49	22.0	22 (45)	375	22.0	252 (67)
15	Yori et al. [53]	2006	Cross-sec.	Peru	Strongyloidiasis	69	23.0*	+	423	23.0*	+
16	Aimpun et al. [59]	2004	Cross-sec.	Belize	Any STH infection	418	19.7*	177 (42)	135	14.1*	119 (88)
17	Lello et al. [60]	2013	Cross-sec.	Zanzibar	Any STH infection	132	+	+	198	+	+
18	Ali et al. [78]	1999	Cross-sec.	Ethiopia	Any STH infection	243	+	112 (46)	39	+	9 (23)
19	Gunawardena et al. [23]	2011	Cross-sec.	Sri Lanka	Any STH infection	549	11.1	238 (43)	1341	11.2	639 (48)
20	Gamboa et al. [24]	2009	Cross-sec.	Argentina	Any STH infection	152	+	+	42	+	+
21	Kurup et al. [58]	2010	Cohort	Saint Lucia	Any STH infection	253	+	193 (76)	301	+	120 (40)
22	Khan et al. [63]	1979	Cross-sec.	India	Any STH infection	27	11.9*	+	32	39.0	+
23	Modjarrad et al. [25]	2005	Cross-sec.	Zambia	Any STH infection	78	29.0	50 (64)	228	31.0	159 (70)
24	Mihrshahi et al. [57]	2009	Cross-sec.	Vietnam	Any STH infection	70	29.7*	70 (100)	296	29.7*	296 (100)
25	Martinez et al. [61]	1961	Cross-sec.	Cuba	Any STH infection	934	4.0*	+	66	4.0*	+
26	Liabsuetrakul et al. [62]	2009	Cross-sec.	Thailand	Any STH infection	190	27.3*	190 (100)	873	27.3*	873 (100)
27	Phiri et al. [56]	2000	Cross-sec.	Malawi	Any STH infection	43	7.2*	+	230	7.2*	
28	Woodburn et al. [64]	2009	RCT	Uganda	Hookworm infection	1112	23.0	1112 (100)	1386	23.0	1386 (100)
29	Traub et al. [65]	2004	Cross-sec.	India	Hookworm infection	141	+	+	187	+	+
30	Tadesse et al. [75]	2005	Cross-sec.	Ethiopia	Hookworm infection	28	11.2*	9 (32)	387	11.2*	135 (35)
31	Pullan et al. [66]	2010	Cross-sec.	Uganda	Hookworm infection	709	+	+	1094	+	+
32	Nmor et al. [67]	2009	Cross-sec.	Nigeria	Hookworm infection	534	8.4*	184 (34)	71	8.8*	278 (63)
33	Lee et al. [68]	2007	Case-control	Brunei	Hookworm infection	18	+	+	100	+	+
34	Jiraanankul et al. [72]	2011	Cohort	Thailand	Hookworm infection	33	+	19 (58)	319	+	191 (60)
35	Ilechukwu et al. [76]	2010	Cross-sec.	Nigeria	Hookworm infection	150	+	+	310	+	+
36	Humphries et al. [73]	2011	Cross-sec.	Ghana	Hookworm infection	116	+	58 (50)	142	+	75 (53)
37	Gutman et al. [69]	2010	Cross-sec.	Nigeria	Hookworm infection	223	12.0*	+	314	12.0*	+
38	Erosie et al. [74]	2002	Cross-sec.	Ethiopia	Hookworm infection	113	10.9*	28 (25)	308	10.9	102 (33)
39	Behnke et al. [71]	2000	Cross-sec.	Mali	Hookworm infection	151	+	+	134	+	+
40	Alemu et al. [70]	2011	Cross-sec.	Ethiopia	Hookworm infection	61	11.0	30 (49)	258	11.0	132 (51)
41	Mukerji et al. [77]	1950	Cross-sec.	India	Hookworm infection	2166	27.0*	595 (27)	3125	24.9*	681 (22)
42	Chongsuvivatwong et al. [16]	1996	Cross-sec.	Thailand	Hookworm infection	100	30.5*	+	92	30.5*	+
43	Bethony et al. [14]	2002	Cross-sec.	China	Hookworm infection	285	31.3*	+	224	31.3*	+
44	Davey et al. [26]	2006	Cross-sec.	Ethiopia	Podoconiosis	248	38.0	122 (49)	1152	38.0	478 (41)
45	Price et al. [82]	1974	Cross-sec.	Ethiopia	Podoconiosis	15977	+	6781 (42)	27; 596	+	6240 (23)
46	Kloos et al. [35]	1992	Cross-sec.	Ethiopia	Podoconiosis	31	+	+	385	+	+
47	Molla et al. [38]	2013	Case-control	Ethiopia	Podoconiosis	460	51.5*	243 (53)	707	4.4*	270 (38)
48	Yakob et al. [83]	2008	Cross-sec.	Ethiopia	Podoconiosis	73	+	+	+	365	+
49	Deribe et al. [81]	2013	Cross-sec.	Ethiopia	Podoconiosis	5253	45	3,045 (58)	124; 706	33	62,056 (50)
50	Muehlen et al. [30]	2006	Cross-sec.	Brazil	Tungiasis	253	16.9*	137 (54)	243	19.2*	147 (60)
51	Njau et al. [80]	2012	Cross-sec.	Kenya	Tungiasis	218	+	+	167	+	+
52	Ugbomoiko et al. [79]	2007	Cross-sec.	Nigeria	Tungiasis	252	18.4*	111 (44)	305	21.1*	147 (48)
53	Thielecke et al. [34]	2013	RCT	Madagascar	Tungiasis	77	26.7	56 (73)	70	25.5	61 (87)

*Age mean reported.

+Missing raw data.

Abrevation: CLM: Cutaneous larva migrans, Cross-sec.: cross-sectional study; RCT: randomized controlled trial.

**Table 3:** A summary of descriptive information ofand study quality by outcome.

		Buruli Ulcer n = 3	CLM n = 1	Leptospirosis n = 7	Strongyloidiasis n = 4	Any STH infection n = 11	Hookworm infection n = 17	Podoconiosis n = 6	Tungiasis n = 4
*Descriptive information*									
Median sample size (range)		230 (136–232)	126	276 (60–1169)	30 (152–492)	330 (59–1890)	1324 (118–5291)	1284 (416–129959)	441 (147–557)
Median % of cases		33	17	36	12	46	10	28	53
Median age	Cases	12	37	32	22	15	17	38	27
	Without disease	13	41	35	22	31	11	38	26
Median % of females	Cases	50	+	40	30	70	30	50	50
	Without disease	50	+	50	60	80	50	40	60
*Study quality*									
Number with cross-sectional survey design (%)		0 (0)	1 (100)	4 (57)	3 (75)	10 (91)	14 (82)	5 (83)	3 (75)
Footwear measured	By self-report	3 (100)	1 (100)	7 (100)	4 (100)	10 (91)	14 (82)	5 (83)	3 (75)
	By observation	0 (0)	0 (0)	0 (0)	0 (0)	1 (9)	3 (18)	1 (17)	1 (25)
Were the following items reported? (%)	Study population	3 (100)	1 (100)	7 (100)	4 (100)	11 (100)	17 (100)	6 (100)	4 (100)
	Selection of participants	2 (67)	1 (100)	5 (71)	3 (75)	5 (45)	14 (82)	4 (67)	4 (100)
	Sample size/power calculation	1 (33)	1 (100)	2 (29)	2 (50)	4 (36)	4 (24)	2 (33)	3 (75)
	Outcome and exposure measurement	3 (100)	1 (100)	7 (100)	4 (100)	9 (82)	15 (88)	5 (83)	3 (75)
	Adjusted estimates for confounding	3 (100)	0 (0)	5 (71)	3 (75)	4 (36)	11 (65)	3 (50)	3 (75)

+Missing data.

**Figure 2:**
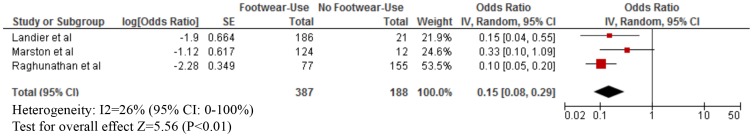
Forest plot of studies showing the association between footwear use and the risk of Buruli ulcer.* *Inverted Log [odds ratio] and standard error (SE) from effect estimate of barefoot exposure: Landier et al [44]. *Log [odds ratio] and SE calculated from raw data: Marston et al [84] and Raghunathan et al [45].

**Figure 3:**
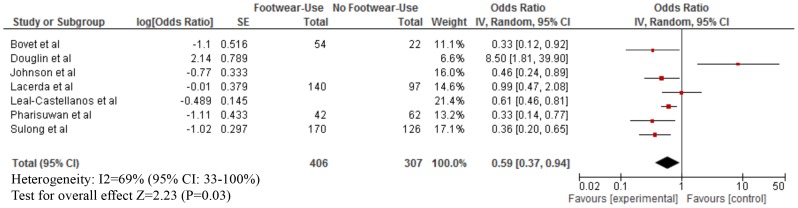
Forest plot of studies showing the association between footwear use and leptospirosis.* *Stratified exposure totals were not given for the following studies: Johnson et al. (N = 648) [46], Leal-Castellanos et al. (N = 1169) [47], Pharisuwan et al. (N = 104) [48], and Sulong et al. (N = 296) [49]. *A 95% confidence interval/standard error (SE) was not available so it was not included in the forest plot: Alarcon et al. (odds ratio: 0.54) [50] *Adjusted effect estimate: Johnson et al. [46] and Sulong et al. [49]. *Inverted Log [odds ratio] and SE from effect estimate of barefoot exposure: Bovet et al. [51], Johnson et al. [46], and Leal-Castellanos et al. [47]. *Log [odds ratio] and SE calculated from raw data: Lacerda et al. [52].

**Figure 4:**
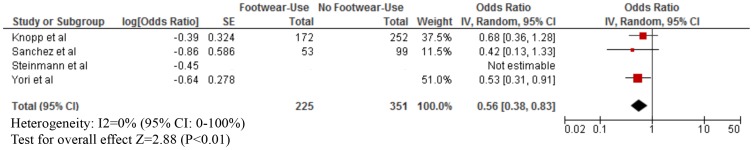
A forest plot of studies showing the association between footwear use and strongyloidiasis.* *Stratified exposure totals were not given for the following studies: Yori et al. (N = 492) [53] and Steinmann et al. (N = 180) [54]. * A 95% confidence interval/standard error (SE) was not available so it was not included in the forest plot: Steinmann et al. (odds ratio: 0.64) [54]. *Adjusted effect estimate: Yori et al. [53]. *Inverted Log [odds ratio] and SE from effect estimate of barefoot exposure: Yori et al. [53]. *Log [odds ratio] and SE calculated from raw data (comparing severe form of illness to chronic infection): Sanchez et al. [55].

**Figure 5:**
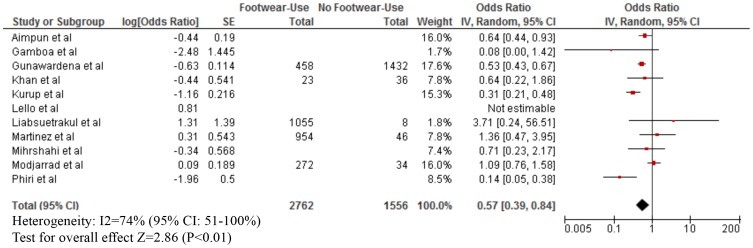
Forest plot of studies showing the association between footwear use and any soil-transmitted helminth infection.+* +Note different x-axis. *Stratified exposure totals were not given for the following studies: Phiri et al. (N = 273) [56], Mihrshahi et al. (N = 366) [57], Kurup et al. (N = 554) [58], and Gamboa et al. (N = 194) [24], Aimpun et al. (N = 553) [59], and Lello et al. (N = 330) [60]. *A 95% confidence interval/standard error (SE) was not available so it was not included in the forest plot: Lello et al. (odds ratio: 0.81) [60]. *Adjusted effect estimate: Phiri et al. [56]and Mihrshahi et al. [57]. *Inverted Log [odds ratio] and SE from effect estimate of barefoot exposure: Phiri et al. [56], Modjarrad et al. [25], Mihrshahi et al. [57], Kurup et al. [58], Gunawardena et al. [23], Gamboa et al. [24]. *Log [odds ratio] and SE calculated from raw data: Martinez et al. [61], Liabsuetrakul et al. [62], and Khan et al. [63].

**Figure 6:**
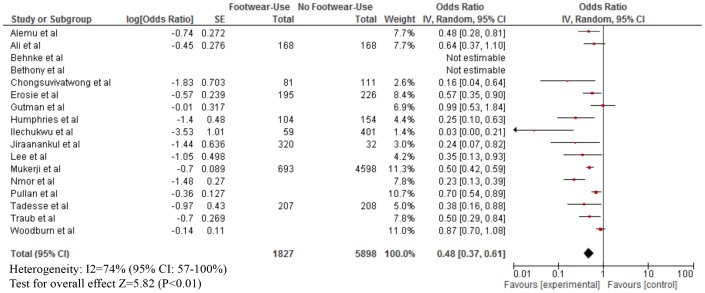
Forest plot of studies showing the association between footwear use and hookworm infection.+* +Note different x-axis. *Stratified exposure totals were not given for the following studies: Woodburn et al. (N = 2498) [64], Traub et al. (N = 328) [65], Pullan et al. (N = 1803) [66], Nmor et al. (N = 978) [67], Lee et al. (N = 118) [68], Gutman et al. (N = 537) [69], Alemu et al. (N = 319) [70] and Behnke et al. (N = 285) [71]. *The magnitude of the odds ratio and the 95% confidence interval/standard error (SE) were not available so it was not included in the forest plot: Behnke et al. [71] (odds ratio for footwear use not significant) and Bethony et al. [14] (footwear use was not significantly associated with hookworm infection). *Adjusted effect estimate: Woodburn et al. [64], Traub et al. [65], Pullan et al. [66], Nmor et al. [67], Lee et al. [68], Jiraanankul et al. [72], Humphries et al. [73], and Gutman et al. [69]. *Inverted Log [odds ratio] and SE from effect estimate of barefoot exposure: Woodburn et al. [64], Traub et al. [65], Pullan et al. [66], Nmor et al. [67], Lee et al. [68], Jiraanankul et al. [72], Humphries et al. [73], Gutman et al. [69], Erosie et al. [74], Alemu et al. [70]. *Log [odds ratio] and SE calculated from raw data: Tadesse et al. [75], Ilechukwu et al. [76], Mukerji et al. [77], Ali et al. [78].

**Figure 7:**
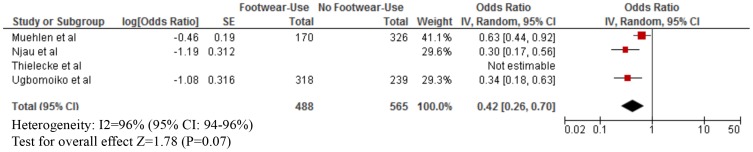
Forest plot of studies showing the association between footwear use and tungiasis.* *Adjusted effect estimate: Ugbomoiko et al. [79]. *Stratified exposure totals were not given for the following studies: Njau et al. [80] (N = 385) and Thielecke et al. [34] (N = 147). *The magnitude of the odds ratio and the 95% confidence interval/standard error (SE) were not available so it was not included in the forest plot: Thielecke et al. [34] (marginal decrease in intensity of infection with footwear use).

**Figure 8:**
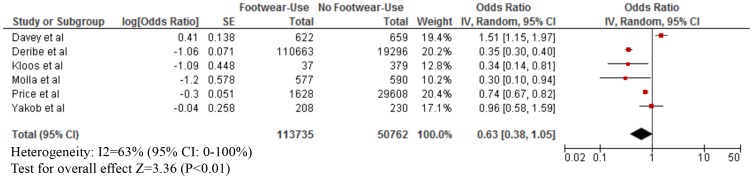
Forest plot of studies showing the association between footwear use and podoconiosis.* *Adjusted effect estimate: Molla et al. [38]. *Inverted Log [odds ratio] and standard error (SE) from effect estimate of barefoot exposure: Deribe et al. [81] and Molla et al. [38]. *Log [odds ratio] and SE calculated from raw data: Price et al. [82], Kloos et al. [35], and Yakob et al. [83].

As shown in Table 3, footwear use was mostly measured by self-report. The median proportion of footwear use was: Buruli ulcer (80% for both cases and those without infection), leptospirosis (cases: 40%; without infection: 50%), strongyloidiasis (cases: 25%; without infection: 40%), any STH infection (cases; 60%; without infection: 97%), hookworm infection (cases: 30%; without infection: 50%), podoconiosis (cases: 55%; without disease: 50%), and tungiasis (cases: 30%; without disease: 60%). Our meta-analyses showed that footwear use was significantly associated with a lower odds of Buruli ulcer (OR = 0.15; 95% CI: 0.08–0.29), CLM (OR = 0.24; 95% CI: 0.06–0.96), leptospirosis (OR = 0.59; 95% CI: 0.37–0.94), strongyloidiasis (OR = 0.56; 95% CI: 0.38–0.83), any STH infection (OR = 0.57; 95% CI: 0.39–0.84), hookworm infection (OR = 0.48; 95% CI: 0.37–0.61), and tungiasis (OR = 0.42; 95% CI: 0.26–0.70) (Figures 2–7). On the other hand, footwear use was not significantly associated with the occurrence of podoconiosis (OR: 0.63; 95% CI: 0.38–1.05), as seen in the forest plot of Figure 8. Estimates of I^2^ varied, including low heterogeneity: strongyloidiasis 0% (95% CI: 0–100%), Buruli ulcer 26% (95% CI: 0–100%); and moderate-to-high heterogeneity: tungiasis 63% (95% CI: 0–100%), leptospirosis 69% (95% CI: 33–100%), any STH infection 74% (95% CI: 51–100%), hookworm infection 74% (95% CI: 57–100%), and podoconiosis 96% (95% CI: 94–96%).

## Discussion

We found that footwear use was significantly associated with a lower odds of Buruli ulcer, CLM, leptospirosis, strongyloidiasis, any STH infection, hookworm infection, and tungiasis, highlighting the important role of footwear use in the prevention of NTDs. No significant association was found between footwear use and podoconiosis. We found no data regarding the use footwear and mycetoma, myiasis, and snakebite. The results presented here have important implications for both policy and practice. Promotion of footwear use should be an important part of selected NTD control strategies.

The significant association between footwear use and the lower odds of Buruli ulcer, CLM, hookworm infection, leptospirosis, and tungiasis are consistent with the mode of transmission of these diseases [16], [20], [29]–[34].The risk factors include presence of skin cuts or abrasions and contact with water, soil, or mud during work or recreational activities if the water is contaminated with human and animal excreta, including rodent urine [33]. Our findings are also consistent with a recent meta-analysis on WASH interventions which included some results regarding footwear use and hookworm infection or any STH infection [7]. This review found that footwear use was significantly associated with a lower odds of hookworm infection (OR 0.29; 95% CI 0.18–0.47) and any STH infection (OR = 0.30; 95% CI 0.11–0.83) [7], as compared to the findings on hookworm infection (OR = 0.48; 95% CI: 0.37–0.61) and strongyloidiasis (OR = 0.56, 95% CI 0.38–0.83) in the current analysis.

We did not find a significant association between footwear use and the risk of podoconiosis. Two issues may explain this: first, podoconiosis is a chronic disease primarily affecting the feet, so reverse causality is likely (when an individual first notices foot or leg swelling, he or she starts wearing shoes), and second, podoconiosis requires a long period of exposure, but assessment of current use of footwear does not reflect previous exposure [35]. Studies comparing podoconiosis patients with healthy controls have found that patients tend to wear footwear more than healthy controls to protect their legs from injury or to conceal the swelling in fear of stigma and discrimination [36], [37]. Other studies have suggested that age at first footwear use would be a more precise indicator of protection than current footwear use [38].

A number of our findings support integrated control strategies of NTDs. Footwear use appears to have a protective effect across multiple NTDs and thus may become an important integrated NTD control measure and should be considered by researchers, program planners, and policy makers. Footwear use interventions also have the potential to enhance sustainability of NTD control programs, similar to improved access to clean water, sanitation, and altered hygiene behavior [1], [5], [7], [39]–[43].Advocacy could be integrated into current efforts such as school health services, and indicators on type and frequency of footwear use could be included in NTD monitoring and evaluation. Initial investments may only be needed to create awareness and demonstrate the practical benefits of footwear use and promote it as a continued behavior. A public-private partnership model similar to that of pharmaceutical companies for population-based chemotherapy could be seen as example to leverage resources with footwear companies. However, future cost-effectiveness studies are needed to fully explore the feasibility and sustainability of these interventions.

We aimed to adhere to the PRISMA statement for the reporting of meta-analysis of observational studies. However, there were several limitations in this systematic review. First, only six out of the 56 included studies specified the type of footwear. Thus, we were unable to explore how the type of footwear may have affected the results. Type and frequency of footwear use may vary regionally due to differences in seasonality, socioeconomic conditions, occupation, and cultural practices. These differences could affect the effectiveness of footwear interventions and practical implementation of related interventions. Only one study was identified for CLM which limited our ability to conduct meta-analysis for this outcome.

Second, there was marked heterogeneity with wide CIs between some studies which may have led to imprecise summary estimates. *I*-squared estimates varied, including low heterogeneity (strongyloidiasis 0% and Buruli ulcer 26%) and moderate-to-high heterogeneity (tungiasis 63%, leptospirosis 69%, any STH infection 74%, hookworm infection 74%, and podoconiosis 96%). This may have been due to the different definitions (e.g., many studies used a questionnaire design without clarifying the type of footwear or consistency of use) or diagnostic methods employed to determine infection or disease across studies. However, we used a random-effects model to calculate summary measures in an attempt to address this heterogeneity. The results using a fixed-effects model did not substantially differ from the random-effects model, indicating only small study biases.

Lastly, most of the studies were observational in nature (e.g., cross-sectional surveys and case-control studies), giving rise to concerns regarding study quality. With cross-sectional surveys, we are unable to reach conclusions about the effect of shoes on the incidence of infection or disease over time and estimates may be confounded by other variables. Only a limited number of studies provided adjusted estimates, often controlling for just a few sociodemographic variables, with potential residual confounding. Case-control studies may be affected by recall bias, depending on how cases recall footwear exposure compared to those without disease. Details on the measurement of footwear use and the presence of NTDs were not always reported which also may have led to biased estimates. Prospective studies specifically designed to look at the effect of footwear use on selected NTDs are needed to answer this research question. RCTs may provide more robust evidence but can be ethically and financially challenging. A recent cluster randomized trial failed to show any association between hookworm infection and footwear use due to contamination [12]. Approaches such as a stepped wedge trial design or a robust cohort study may offer more feasible solutions.

### Conclusions

NTDs have multiple routes of transmission and a single intervention alone is unlikely to completely interrupt transmission. Little attention has focused on personal preventive measures to reduce exposure to infection, such as the use of footwear. Our findings provide evidence that footwear use could help prevent a range of different NTDs, including Buruli ulcer, leptospirosis, CLM, tungiasis, any STH infection, strongyloidiasis, and hookworm infection. Although prospective data are still needed to explore the effect of footwear use on the incidence of NTDs over time, these findings support the integrated control strategies of NTDs that include footwear use. Initial investments are required to create awareness and demonstrate the practical benefits of footwear use and promote it as a continued behavior. There may also be a need to provide footwear to particular at-risk groups (e.g., school-aged children for STH infections), and a similar public-private partnership model to that used with pharmaceutical companies for large-scale preventive chemotherapy might be applied to leverage resources with footwear companies. However, future cost-effectiveness studies are needed to fully explore the feasibility and sustainability of these interventions.

## Supporting Information

Checklist S1 PRISMA checklist.
(DOC)

Protocol S1 Study protocol.
(DOC)
