# Supplementary material for: Association between Footwear Use and Neglected Tropical Diseases: A Systematic Review and Meta-Analysis
Source: PLoS Negl Trop Dis. 2014 Nov 13;8(11):e3285. doi: 10.1371/journal.pntd.0003285 (PMC4230915; doi:10.1371/journal.pntd.0003285)
Supplement: Protocol S1 — Study protocol. (DOC) [file pntd.0003285.s002.doc]

**NTDs and Shoes Lit Review 2012 Protocol**

1. Title:

Does shoe wearing decrease the prevalence or incidence of selected neglected tropical diseases in endemic settings? A systematic review

1. Authors:

Kebede Deribe

Sara Tomczyk

Gail Davey

1. Contributing Citations:

Prof. Simon Brooker

Prof. David Warrell

Prof. Peter Hotez

Dr. Miriam Eddyani

1. Sources of Support:

Author salaries, Brighton and Sussex Medical School (BSMS) Library

1. Protocol Published:

November 2012

1. Background Objective

The objective of this review is to assess the effects of shoe wearing on the prevalence or incidence in selected (10) neglected tropical diseases in children and adults in endemic communities, including buruli ulcer, cutaneous larva migrans, leptospirosis, madura foot, myiasis, podoconiosis, snakebite, soil-transmitted helminths, strongyloidiasis, and tungiasis.

1. Selection Criteria
   1. Study Designs: All intervention and observational studies (Observational studies are being included because limited trials may exist to answer this research question.)
   2. Study Settings: All relevant endemic settings
   3. Participants: Men, women and children
   4. Interventions: Shoe-wearing (including all types of footwear)
   5. Outcome Measures: The effect of shoe-wearing on the prevalence, incidence or intensity of infection of disease including all NTDs that could be transmitted from walking barefoot
   6. Publication Status: All published data, abstracts and grey literature
   7. Data were Published: All dates
   8. Language of Published Articles: All languages
2. Search Strategy

The following databases will be searched for manuscripts: Medline, Embase, Cochrane, Web of Science, CINAHL Plus, and Popline. The keywords searched can be seen in Table I. The reference lists of all manuscripts identified from the database search will be reviewed for additional citations. Experts in selected NTD areas will also be contacted for further citation recommendations relevant to the research question.

1. Methods of Review
   1. Authors ST and KD will perform the database search and the hand-searching of the reference lists. Additional support from author GD and her institution BSMS will be given to help obtain full manuscripts of the identified citations. ST, KD, and GD will contacts the experts in selected NTD areas for additional citation recommendations.
   2. KD and ST will screen identified citations independently according to the selection criteria including rapid appraisal of full manuscripts. If no consensus is reached between KD and ST, GD will review. Excluded articles and reasons for exclusion will be documented according to Table II (will be developed into a flowchart).
   3. A standardised data extraction form (e.g. excel) will be developed based on Preferred Reporting Items for Systematic Reviews and Meta-Analyses (PRISMA) and piloted. It will include:
      1. Study identifier
      2. Study characteristics: Study population (age, sex, setting), sample size, selection, type of shoe, frequency of shoe-wearing, length of follow-up, study design, publication status/year, language, source
      3. Study outcome measures
      4. Study quality: According to a selected checklist
   4. KD and ST will extract data independently according to the data extraction form. If no consensus is reached between KD and ST, GD will review.
   5. KD and ST will assess potential publication bias including a funnel plot.
   6. KD and ST will analyse the data including a forest plot to show the individual effect estimates of included studies. A meta-analysis will be considered if possible.
   7. KD and ST will analyse the following sensitivity analyses if possible:
      1. Exclusion of studies likely to be low quality according to the performed checklist
      2. Limited to published data
   8. KD, ST, and GD will interpret the results including
      1. Review of findings including study quality
      2. Consistency and plausibility of findings
      3. Explanation for potential study heterogeneity
      4. Strengths of the review
      5. Limitations of the review
      6. Application of study findings to public health practice; Generalization
      7. Recommendations to improve conduct of studies and areas for future research
      8. The reporting will follow PRISMA.
2. Acknowledgements

The support received from BSMS library and the experts in selected NTD areas for additional citation recommendations should be acknowledged.

1. Conflicts of Interest

Author ST and KD receive salary support from TOMS Shoes.

1. Tables

Table I. Search Strategy for Identifying Manuscripts

| # | Condition | Term #1 | Term #2 |
| --- | --- | --- | --- |
| 1 | All NTDs | exp shoes OR shoe* OR footwear* OR boots OR sandals OR footgear | exp Neglected Diseases OR neglected tropical disease* OR NTD*OR exp tropical disease |
| 2 | Buruli Ulcer++ | exp shoes OR shoe* OR footwear* OR boots OR sandals OR footgear | exp Buruli Ulcer OR exp Mycobacterium ulcerans OR exp mycobacterium Infections, nontuberculous OR buruli ulcer* OR mycobacterium ulceran* OR Bairnsdale ulcer OR Daintree ulcer |
|  |  | Exp primary prevention | exp Buruli Ulcer OR exp Mycobacterium ulcerans OR exp mycobacterium Infections, nontuberculous OR buruli ulcer* OR mycobacterium ulceran* OR Bairnsdale ulcer OR Daintree ulcer |
| 3 | Podo-coniosis++ | exp shoes OR shoe* OR footwear* OR boots OR sandals OR footgear | Podoconiosis OR non?filarial elephantiasis OR mossy foot |
|  |  | Exp primary prevention | Podoconiosis OR non?filarial elephantiasis OR mossy foot |
| 6 | STHs++ | exp shoes OR shoe* OR footwear* OR boots OR sandals OR footgear | Soil-transmitted helminth* OR soil transmitted helminth* OR intestinal worm* OR exp helminth OR exp Helminthiasis |
|  |  | Exp primary prevention | Soil-transmitted helminth* OR soil transmitted helminth* OR intestinal worm* OR exp helminth OR exp Helminthiasis |
| 7 | Hookworm only++ | exp shoes OR shoe* OR footwear* OR boots OR sandals OR footgear | Exp hookworm infections OR exp ancylostomatoidea OR exp ancylostoma OR necator |
|  |  | Exp primary prevention | Exp hookworm infections OR exp ancylostomatoidea OR exp ancylostoma OR necator |
| 8 | Strongy-loidiasis++ | exp shoes OR shoe* OR footwear* OR boots OR sandals OR footgear | Strongyloid* OR exp strongyloidesstercoralis OR exp Stronyloidiasis OR exp strongyloides OR round?worm |
|  |  | Exp primary prevention | Strongyloid* OR exp strongyloidesstercoralis OR exp Stronyloidiasis OR exp strongyloides OR round?worm |
| 9 | Cutaneous Larva Migrans | exp shoes OR shoe* OR footwear* OR boots OR sandals OR footgear | Cutaneous Larva Migrans* OR creeping eruption OR ground itch OR sandworm* OR plumber’s itch OR zoonotic hookworm OR ancylostomabraziliense OR uncinariastenocephalaOR ancylostomacaninum |
|  |  | Exp primary prevention | Exp Larva Migrans OR Cutaneous Larva Migran* OR creeping eruption OR ground itch OR sandworm* OR ancylostomabraziliense OR uncinariastenocephalaOR ancylostomacaninum OR exp ancylostoma |
| 10 | Lepto-spirosis | exp shoes OR shoe* OR footwear* OR boots OR sandals OR footgear | Exp leptospirosis OR exp weil disease OR canicola fever OR canefield fever OR nanukayami fever OR Rat Catcher’s Yellows OR Fort Bragg Fever OR black jaundice OR Pretibial fever OR exp Leptospira OR Icterohemorrhagic fever OR Swineherd's disease OR Rice-field fever OR Cane-cutter fever OR Swamp fever OR Mud fever OR Hemorrhagic jaundice OR Stuttgart disease |
|  |  | Exp primary prevention | Leptospirosis OR weil’s syndrome OR weil disease OR canicola fever OR canefield fever OR nanukayami fever OR 7-day fever OR Rat Catcher’s Yellows OR Fort Bragg Fever OR black jaundice OR Pretibial fever OR Leptospira OR Icterohemorrhagic fever OR Swineherd's disease OR Rice-field fever OR Cane-cutter fever OR Swamp fever OR Mud fever OR Hemorrhagic jaundice OR Stuttgart disease |
| 11 | Tungiasis | exp shoes OR shoe* OR footwear* OR boots OR sandals OR footgear | Exp tunga OR Tungapenetrans OR jigger* OR sandflea OR exp Tungiasis OR Pico OR chigoe flea OR suthi |
|  |  | Exp primary prevention | Exp tunga OR Tungapenetrans OR jigger* OR sandflea OR exp Tungiasis OR Pico OR chigoe flea OR suthi |
| 12 | Myiasis | exp shoes OR shoe* OR footwear* OR boots OR sandals OR footgear | Exp myiasis OR dermatobiahominis OR chrysomabezziana OR cordylobiaanthropophaga OR flystrike OR blowfly strike OR fly-blown |
|  |  | Exp primary prevention | Exp myiasis OR dermatobiahominis OR chrysomabezziana OR cordylobiaanthropophaga OR flystrike OR blowfly strike OR fly-blown |
| 13 | Snakebite ++ | exp shoes OR shoe* OR footwear* OR boots OR sandals OR footgear | Exp Snake bite OR exp antivenins OR snakebite* OR exp venoms OR envenoming OR snake poison |
|  |  | Exp primary prevention | Exp Snake bite OR exp antivenins OR snakebite* OR exp venoms OR envenoming OR snake poison |
| 14 | Madura Foot | exp shoes OR shoe* OR footwear* OR boots OR sandals OR footgear | Madura Foot OR exp mycetoma OR eumycetoma* OR mycetomapedis OR actinomycetoma* |
|  |  | Exp primary prevention | Madura Foot OR exp mycetoma OR eumycetoma* OR mycetomapedis OR actinomycetoma* |

+Search strategy for Ovid Medline database. A modified search strategy was used for the remaining databases according to the available search terms.

++NTDs in WHO list.

Table II. Identification and Screening of Manuscripts

| Disease | # Identified | # Selected upon Screening | Reasons for Exclusion |
| --- | --- | --- | --- |
| Buruli Ulcer |  |  |  |
| CLM |  |  |  |
| Leptospirosis |  |  |  |
| Madura Foot |  |  |  |
| Podoconiosis |  |  |  |
| Snakebite |  |  |  |
| STHs |  |  |  |
| Strongyloidiasis |  |  |  |
| Tungiasis |  |  |  |
